# Imepitoin is well tolerated in healthy and epileptic cats

**DOI:** 10.1186/s12917-017-1087-3

**Published:** 2017-06-12

**Authors:** Odilo Engel, Thilo von Klopmann, Arianna Maiolini, Jessica Freundt-Revilla, Andrea Tipold

**Affiliations:** 1 Boehringer Ingelheim Vetmedica GmbH, Binger Str. 173, 55216 Ingelheim am Rhein, Germany; 2 Tierklinik Hofheim, Katharina-Kemmler-Str. 7, 65719 Hofheim, Germany; 3 University of Veterinary Medicine, Department of Small Animal Medicine and Surgery, Bünteweg 9, 30559 Hannover, Germany; 4 Center for Systems Neuroscience, Hannover, Germany

**Keywords:** Epilepsy, Cat, Imepitoin, Clinical trial

## Abstract

**Background:**

Epilepsy in the cat is a serious medical condition. To date there are no licensed treatments for feline epilepsy and no well-controlled clinical studies on the efficacy or safety of antiepileptic drugs in cats. The aim of this study was to collect tolerability ﻿data﻿ and first exploratory efficacy data of imepitoin in both healthy and epileptic cats.

**Results:**

In two tolerability studies, 30 healthy cats received imepition twice daily in doses of 0, 30, 40 or 80 mg/kg bodyweight for 30 days. No serious adverse events were observed in any of the dose groups. In the imepitoin treated groups, emesis was observed in some animals temporarily and intermittently mainly in the second and third weeks of treatment.

In a small, single-arm, open label, uncontrolled clinical trial eight cats suffering from idiopathic epilepsy were treated with imepitoin twice daily at doses of 30 mg/kg bodyweight for 30 days. Four of these cats (50%) achieved seizure freedom for at least 8 weeks under treatment. Adverse events, mostly lethargy, decreased appetite and emesis, were often mild and transient.

**Conclusion:**

In summary, imepitoin was well tolerated in healthy and epileptic cats and showed in a pilot trial indication for efficacy in treating feline epilepsy.

## Background

Epileptic seizures are the most common neurologic problem encountered in small animal medicine [1]. When possible, the underlying cause of occurring seizures is identified and treated. If the underlying cause cannot be identified or is untreatable, treatment involves the administration of antiepileptic drugs in an attempt to control seizure frequency and/ or seizure severity [2]. Appropriate treatment can be especially difficult in cats since to date there are no licensed treatments for feline epilepsy and no well-controlled clinical studies on the efficacy or safety of antiepileptic drugs in cats [3].

Whereas the etiology and diagnosis of epilepsy show similarities between dogs and cats, treatment with antiepileptic drugs (AEDs) in cats is different than in dogs [3, 4].

Benzodiazepine-like agents act through positive allosteric modulation of gamma-aminobutyric acid_A_ (GABA_A_) receptors, potentiating the inhibitory effects of GABA in inducing Cl^−^ currents, leading to hyperpolarisation of neurons and inhibitory effects on transmission [5]. Benzodiazepines such as diazepam or clonazepam are considered highly efficacious in treating many forms of epilepsy. However, these benzodiazepines act as full agonists of GABA_A_ receptors, and long-term treatment is associated with loss of efficacy (tolerance) and development of physical dependence in both dogs and humans [6, 7].

In cats, oral diazepam has a longer elimination half-life (15–20 h) than in dogs (3–4 h) and cats do not develop functional tolerance to the drug in contrast to other species, including rat, mouse, dog and human [8, 9]. Along with non-fatal adverse events like sedation, polyuria and polydipsia, diazepam has been linked to potentially fatal idiosyncratic hepatotoxicosis, hepatic necrosis and liver failure in cats [10]. Consequently, diazepam is often not considered a viable maintenance oral anticonvulsant option for cats [4, 11]. For the same reason other benzodiazepines like clorazepate are not recommended [12].

Imepitoin is a low-affinity partial agonist acting at the benzodiazepine recognition site of the GABA_A_ receptor and was specifically developed for the treatment of canine epilepsy [13]. Anticonvulsant activity and safety in dogs were evaluated in several clinical and laboratory studies, and on this basis imepitoin was licensed in the European Union and in other countries to reduce the frequency of generalized seizures due to idiopathic epilepsy in dogs [14]. Imepitoin is now considered a first-line treatment option in dogs with idiopathic epilepsy [15].

As cats react differently to some antiepileptic drugs than dogs, an extrapolation from dog data to the feline situation is not possible. Since other drugs acting at the benzodiazepine recognition site on GABA_A_ receptors, appear to be efficacious in treating epileptic cats, we hypothesized that imepitoin maintains the good anticonvulsant efficacy of benzodiazepines in cats, but as partial low-affinity agonist will demonstrate the tolerability seen in other species. The aim of these studies was to examine the tolerability of imepitoin in both healthy and epileptic cats, and to obtain initial data on efficacy as a pilot trial for further power analyses.

## Methods

### Experimental design

Three independent trials were conducted. Two tolerability studies were performed in healthy laboratory cats and one efficacy study was conducted in epileptic cats. In one of the tolerability studies pharmacokinetics were also evaluated. All three studies utilized different doses of imepitoin.

Study 1: In the first randomized, controlled, blinded GLP (good laboratory practice) laboratory study, six cats (3 male, 3 female) received orally 30 mg/kg imepitoin twice daily over 30 days. Six untreated cats (3 male, 3 female) served as controls.

Study 2: The aim of the second, randomized, controlled, blinded GLP laboratory study was to examine safety in higher doses of 40 mg/kg and 80 mg/kg twice daily, while placebo-treated animals served as control. Each of the three parallel groups consisted of six cats (3 male, 3 female), and the treatment duration was 30 days.

Study 3: To evaluate tolerability under field conditions in epileptic cats, a small, single-arm, open label, uncontrolled clinical pilot trial was performed in two centers. In addition, efficacy parameters were assessed. Eight cats suffering from idiopathic epilepsy (TIER II confidence level [16]) were treated with 30 mg/kg imepitoin twice daily for 8 weeks.

### Medication

Imepitoin (Pexion^®^, Boehringer Ingelheim, Ingelheim, Germany) was used in 100 mg or 400 mg tablets, each divisible in two equal parts. For placebo, visually identical tablets without active ingredient were used. In the laboratory studies, medication was provided twice daily, approximately 12 h apart, using a tablet applicator (BUSTER; Jergen Kruuse A/S, Langeskov, Denmark). In the clinical trial, owners provided tablets twice daily in the morning and in the evening.

### Laboratory trials in healthy cats

Intact domestic short hair cats (Liberty Research Inc., Waverly, NY, USA), aged one to 3 years, were kept under a 12 h light/dark cycle at room temperature (15 °C – 26 °C). Drinking water was offered ad libitum, and food once daily. Food and water consumption was monitored once daily during the study period and at baseline. Environmental enrichment (toys, shelves, etc.) was provided to all cats. In the first study (30 mg/kg), cats were housed in groups (3 cats of same gender), while in the second study (40/80 mg/kg) cats were kept individually for the study period.

Cats were observed daily for occurrence of adverse events and general wellbeing, and a physical examination was performed before treatment start, at day 7 (only in 40/80 mg/kg study), day 14 and day 30. This included a general check of all body systems; body temperature measurement, behavior and nervous system evaluation, cardiovascular and respiratory assessment, and visual examination of the eyes. Body weight was measured once a week.

Blood samples were taken for hematology and clinical chemistry 1 day before first dosing, at day 15 in study 1 (30 mg/kg study) and in all cats at day 30. An automated complete blood count, including differential blood count (ADVIA hematology system, Siemens Healthcare Diagnostics, Eschborn, Germany) and coagulation assessment was performed. Clinical chemistry included a comprehensive metabolic panel (study 1: HECKMAN Synchron CX7; Heckman Coulter, Inc., Fullerton, USA or study 2: KONELAB 30i, Thermo Fisher Scientific, Dreieich, Germany) and additionally cholesterol, triglycerides, phosphate and glutamate dehydrogenase (GLDH). Urine was analyzed with test strips (study 1: Urispec® 9+ Leuko Plus; Henry Schein Inc., Melville, USA or study 2: Combur 9® test, Roche Diagnostics, Mannheim, Germany) and microscopically 1 day before dosing, at day 15 (only study 1) and at day 30. In the 30 mg/kg study, pharmacokinetics were assessed at day 1, 15 and 29 collecting blood 0 h (i.e. prior to treatment) and 30 min, 1, 3, 6 and 24 h after the first treatment. To obtain at least 100 μL K3EDTA-plasma per animal and sampling time, sufficient whole blood was collected from the vena cephalica, saphena or jugularis. Pharmacokinetic parameters were calculated with Phoenix WinNonlin (Version 6.4; Pharsight/Certara, St. Louis MO 63101; USA) applying a noncompartmental analysis. To validate an adequate exposure to the test substance, in the 40 and 80 mg/kg study, blood was collected at day 1 and 28 at timepoints 0, 1, 3 and 24 h after first dosing.

### Clinical trial

The study was conducted as a multicenter clinical field trial, observing cats with newly diagnosed idiopathic epilepsy for 8 weeks of treatment with 30 mg/kg imepitoin twice daily. If cats stayed on imepitoin monotherapy after this 8 weeks observation period, investigators attempted to follow the cat for as long as possible.

Privately owned cats with epilepsy were included, if they had a history of at least two generalized seizures or focal seizures in the last 2 weeks prior to inclusion, with at least two of the following signs: drooling, facial twitching, tremor, rapid running, mydriasis, hypersalivation, disorientation, or impaired consciousness. Furthermore, the minimum age had to be 9 months and a signed Owner-Informed-Consent had to be obtained. All cats had an MRI scan to rule out structural intracranial lesions as cause for seizures (TIER II [16]). Owners were asked to describe the seizures of their cat, and the investigator graded them as focal or generalized.

A cat was excluded, if it had been on oral antiepileptic treatment for more than 3 consecutive days within the last 6 months prior to inclusion, was treated with antiepileptic drugs within the last 24 h prior to inclusion, was known or suspected to be pregnant or lactating, had a known or suspected concomitant disease that may be accompanied by or result in neurological symptoms (e.g. renal or liver failure, diabetes mellitus) which might interfere with interpretation of the study results, or had a life-threatening disease which may prevent completion of the study (e.g. congestive heart failure).

#### Safety evaluation

At the end of the study, the investigator conducted a clinical examination to determine the general health status of the cat and asked the owners for their observations. In addition, owners were asked to note observations of possible adverse events in the epilepsy seizure diary or to contact the investigator immediately, if adverse events occurred. All adverse events were recorded and classified according to the Veterinary Dictionary for Drug Related Affairs (VeDDRA).

#### Clinical efficacy assessment

During the study, owners were asked to observe their cat for occurrence of seizures and to keep a record of the occurrence of seizures (both focal and generalized) in a patient diary. After 8 weeks, the investigator reviewed the patient diary with the owner and recorded the total number of seizures occurring during the study period, the time to first seizure and the characteristics of the seizures.

The primary measure of efficacy was the achievement of seizure freedom [17]. Animals with at least 8 subsequent weeks of seizure freedom and no reported seizure afterwards were counted as treatment success, at least 50% reduction in seizure frequency as partial success and no sufficient improvement or lost to follow-up as treatment failure. Seizures per individual treatment week were recorded for each patient and plotted over time, modifying a proposal for human epilepsy trials [18]. In addition, monthly seizure frequency (MSF) before and during treatment was calculated by dividing the number of seizures by the number of weeks under observation and multiplying the result by 4. The response ratio, defined as the ratio [(*T* − *B*)/(*T* + *B*)] × 100 where *B* = Baseline Seizure Rate and *T* = Treatment Seizure Rate, was used to obtain a ‘symmetrized’ percent change of seizure activity [19]. For all calculations, generalized and partial seizures were taken into account.

### Statistics

According to the nature of the data and the purpose of the study, we applied appropriate descriptive statistics. Data from the clinical trial were analyzed by two-sided Wilcoxon matched-pairs signed rank test using GraphPad Prism 6.05 (GraphPad Software, Inc., La Jolla, CA, USA; conventionally *p* < 0.05 was considered significant).

## Results

### Imepitoin was well tolerated by healthy cats

No serious adverse events were observed in any of the dose groups. Behavioural changes or sedation were not noted during the course of the study. Repeated oral administration of imepitoin to clinically healthy male and female cats at doses of 30, 40 and 80 mg imepitoin/kg body weight twice daily for 30 days, led to occasional emesis after at least 1 week of treatment. Emesis was intermittently observed until test day 22 of treatment, possibly indicating a transient effect. However, as emesis was more commonly observed in verum groups, it is considered test-item related. Salivation was the most frequent finding in the 40 and 80 mg/kg groups, while it was observed in only one placebo cat.

At the highest doses (40 and 80 mg/kg), a slight reduction in food consumption was observed, especially at the beginning of the study, with subsequently slightly lower body weights compared to controls. No effect on body weight or food consumption was observed in the placebo and 30 mg/kg group.

Urine analysis revealed no clinically evident effects of the treatment. Haematology and clinical chemistry revealed no relevant changes. No changes in liver enzymes or other indicators of hepatic malfunction were observed.

### Pharmacokinetics of imepitoin in cats is similar to dogs

Pharmacokinetics was evaluated in the 30 mg/kg experiment, and plasma samples were taken at 0, 0.5, 1, 3, 6 and 24 h post first treatment (second treatment 8-12 h after first treatment). Therefore, no sampling points were taken at the absorption and distribution phase of the second dose and the pharmacokinetic evaluation was based on the plasma concentration data of the first absorption/distribution phase (0, 0.5, 1, 3, 6 h post dose) and the 24 h value (end of the second absorption/distribution phase). No relevant gender effect was observed, and accordingly male and female data (*n* = 3 per gender) were combined for the pharmacokinetic analysis.

**Fig. 1:**
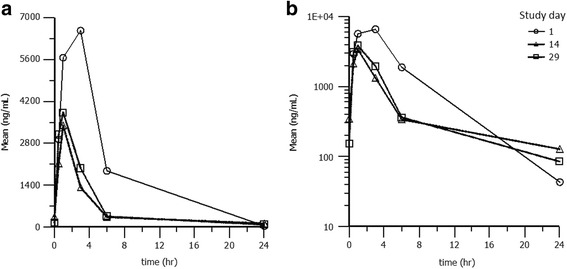
**a** Mean Imepitoin plasma concentration over time on day 1, 14, and 29 (linear scale) **b** Logarithmic scale of mean Imepitoin plasma concentration over time on day 1, 14, and 29

After 14 and 29 days of dosing a decrease in Cmax (about 50%) and an about 60% reduction in exposure (AUC) compared with day 1 data was observed (Table 1; Fig. 1). The observed vomitus may have led to the fact that individual animals had not received the full target dose on days 14 and 29 which consequently resulted in a lower systemic exposure and lower Cmax values and may have been the reason for the observed differences.

**Table 1:** Pharmakokinetic parameters of imepitoin in healthy cats

	Day								
	1			14			29		
	Cmax	AUClast	Tmax	Cmax	AUClast	Tmax	Cmax	AUClast	Tmax
Dose 30 mg/kg	9990	39,640	3.0	2940	11,720	1.0	1590	6932	3.0
	5180	35,870	1.0	1840	7615	1.0	5600	47,560	1.0
	5980	46,780	3.0	1910	9726	0.5	934	4242	0.5
	7220	35,030	3.0	4380	9883	1.0	1400	4115	0.5
	6830	47,970	1.0	6540	22,620	1.0	6300	26,190	1.0
	5880	35,790	3.0	3240	15,850	1.0	7500	20,800	1.0
N	6	6	6	6	6	6	6	6	6
Mean	6850	40,200	2.3	3480	12,900	0.92	3890	18,300	1.2
SD	1700	5810	1.0	1770	5510	0.20	2900	17,000	0.93

### Safety under field conditions

A total of 8 cats with newly diagnosed epilepsy with no known underlying cause were recruited and received at least a single dose of the appropriate drug treatment. One cat was lost to follow-up, as neither the owner nor the referring veterinarian provided further information.

Represented breeds were Domestic Shorthair (DSH, *n* = 2) and European Shorthair (ESH, *n* = 6). Mean age was 6.3 years (SD 5.8; Median 4; Range 1 to 15). Half of the cats were female (*n* = 4), and all cats were neutered (*n* = 8). Mean body weight at inclusion was 4.5 kg (SD 0.76 Range 3.0–5.0 kg). The mean initial dose was 27.92 mg/kg (SD 5.41; Median 30 mg/kg) twice daily. Four cats had concomitant diseases, and one of these cats had multiple diseases (hyperthyroidism, fibrosarcoma, toothache and developed feline infectious peritonitis after the observation period). In the other cats the diseases were not considered to likely cause neurological signs.

At least one adverse event was observed in five of the seven evaluable cats. Most adverse events reported were mild and occurred transiently or intermittently. Lethargy was observed in two cases, which resolved completely by lowering the dose to 20 mg/kg twice daily and keeping the dose at this level. Two cats showed emesis intermittently, and decreased appetite was reported two times. In addition, ataxia, polydipsia, increased appetite, increased salivation, decreased appetite and decreased drinking were reported one time each.

### Efficacy under field conditions

**Fig. 2:**
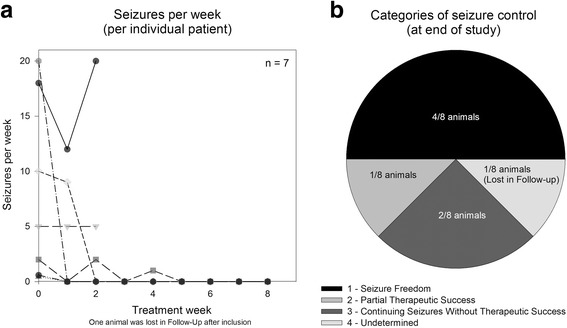
**a** Seizures per treatment week (each line represents one individual patient). Two cats had an identical course of disease (1 seizure/week at baseline). **b** 4 / 8 animals were classified as seizure-free and 1 / 8 had a partial therapeutic success with decreasing frequency of seizures over time, while 2 / 8 continued seizuring without therapeutic success and one was considered undetermined due to lost to follow-up

In total, 4 out of 8 cats achieved seizure freedom at the end of the study, while 1/8 experienced a partial therapeutic success, and 2/8 continued seizuring without therapeutic success. One cat was lost to follow-up, and accordingly 1/8 was considered undetermined and accordingly treatment failure (Fig. 2).

The monthly seizure frequency at baseline was 57.71 ± 73.77 seizures/month (mean ± SD; median 20; range 2 to 200), which reduced significantly under treatment to 19.43 ± 37.15 seizures/month (median 1.5; range 0 to 100; *p* = 0.0313 in Wilcoxon matched-pairs signed rank test compared to baseline). The response ratio was −68.42 ± 38.52 (mean ± SD). Out of the four cats which achieved seizure freedom at the end of the 8 week observation period, the mean reported time of seizure freedom until they were lost to follow up in the extended follow-up period was 16.4 weeks (SD 11.4; range 8.0–30.4 weeks). In none of these cats further seizures were reported until lost to follow-up.

One cat had 3 seizures during the first 4 weeks of treatment, and had no further seizures for the next 5 weeks. As it was lost to follow-up then and did not meet 8 weeks of seizure freedom, it was classified partial success.

In the two cats that continued seizuring without therapeutic success, one cat had continuous seizure activity during the first 2 weeks of imepitoin treatment, so phenobarbital (1 mg/kg twice daily, serum level 34.5 μmol/ml) was added to imepitoin in the third week after inclusion. Seizure frequency decreased during the following weeks, and from week 7 after inclusion onwards, no further seizures were observed. Seizure freedom remained for 4 weeks. Afterwards no information was available.

Following an initial improvement in the first week, the other cat experienced highly frequent focal seizures in the second week of imepitoin treatment. Subsequently, the owners wished to change treatment, and levetiracetam (20 mg/kg 3 times daily) was added to imepitoin treatment. A slight improvement in seizure frequency was observed, however even with increasing the dose of levetiracetam (to 30 mg/kg 3 times daily), sufficient seizure control was not achieved. In week six after inclusion, imepitoin was gradually replaced by phenobarbital. After switching to phenobarbital and levetiracetam, no information on the further course of disease was available.

## Discussion

This is the first report demonstrating the safety and first exploratory efficacy data of imepitoin in cats with epilepsy, and it describes one of the rare prospective clinical trials in feline epilepsy.

Usually, laboratory studies evaluating the safety of veterinary drug candidates end with a complete necropsy. In contrast, new drugs for humans are tested during initial Phase I clinical trials in a small number of healthy volunteers in escalating doses to analyze safety, tolerance and pharmacokinetics of the given drug. Afterwards, during Phase II, the drug is given for the first time to patients to analyze the therapeutic effect of the dose, and then in Phase III the efficacy of the new drug is evaluated in clinical practices. Based on the extensive toxicological data already available for imepitoin [13], we more or less followed the first two phases of human new drug development.

In the first phase, we applied escalating doses of 30 mg/kg, 40 mg/kg and 80 mg/kg imepitoin twice daily to different groups of healthy cats. No serious adverse events were observed in any of the dose groups. In the imepitoin treated groups, emesis was observed in some animals temporarily and intermittently mainly in the second and third weeks of treatment. No signs of sedation were observed. All hepatic parameters assessed revealed no influence of imepitoin on the liver, however no pathology was performed due to the aforementioned reasons. We hypothesize that emesis might be reduced by suitable application protocols like reduction of stress, application with food, or use of antiemetics. However this remains to be examined in dedicated future studies.

In the second phase, the good safety profile of imepitoin in healthy cats was confirmed under field conditions in epileptic cats, and - in contrast to benzodiazepines or other antiepileptics [8] - we did not observe “cat-specific” reactions to imepitoin compared to other species. The pharmacokinetic profile appeared to be similar to dogs [20]. It appears likely, that vomiting accounted for the observed decrease in exposure over the course of the study.

In general, the prognosis of epilepsy of unknown cause in cats is regarded as good if appropriately treated [21]. In this prospective clinical trial, seizure freedom was achieved in 50% of cats, however the follow-up period was relatively short for a definitive conclusion on long-term outcome [17]. Clinical experiences with phenobarbital resulted in seizure freedom in about 40–50% of patients treated [22–24]. A similar rate was reported for bromide, however associated with more adverse events including idiosyncratic allergic pneumonitis [25]. The only available data on levetiracetam as add-on therapy to phenobarbital reported 25–30% seizure freedom [26]. Our study demonstrated a rate of seizure freedom with imepitoin treatment similar to that previously reported for phenobarbital in cats. Evaluating individual seizure incidences per week revealed that seizures may not disappear immediately, but appear to decrease gradually during the first weeks of treatment.

Despite all efforts to include only cats with idiopathic epilepsy, one of the cats included in the field efficacy study suffered from Feline Infectious Peritonitis, which may be a cause of seizures [27]. This cat responded well to antiepileptic treatment with imepitoin.

## Conclusions

To date there are no licensed treatments for feline epilepsy and no well-controlled clinical studies on the efficacy or safety of antiepileptic drugs in cats. Based on the data presented, imepitoin appears to be a potential candidate for treatment of epilepsy in cats. In all three studies treatment with imepitoin was well tolerated in cats. The results suggest a potential therapeutic effect, however the main limitation of the clinical trial is the small sample size, and accordingly no definitive conclusions on efficacy can be made. Further larger studies are needed to confirm the results of this initial pilot study.

## Abbreviations

AED: Antiepileptic drug
GABA: γ-Amino butyric acid
GLP: Good Laboratory Practice
MRI: Magnetic resonance imaging
MSF: Monthly seizure frequency
SD: Standard Deviation
